# A construct of adipose-derived mesenchymal stem cells—laden collagen scaffold for fertility restoration by inhibiting fibrosis in a rat model of endometrial injury

**DOI:** 10.1093/rb/rbad080

**Published:** 2023-09-07

**Authors:** Yangyang Dai, Liaobing Xin, Sentao Hu, Shiqian Xu, Dong Huang, Xiaoying Jin, Jianmin Chen, Rachel Wah Shan Chan, Ernest Hung Yu Ng, William Shu Biu Yeung, Lie Ma, Songying Zhang

**Affiliations:** Assisted Reproduction Unit, Department of Obstetrics and Gynecology, Sir Run Run Shaw Hospital, Zhejiang University School of Medicine, Hangzhou 310016, China; Key Laboratory of Reproductive Dysfunction Management of Zhejiang Province, Hangzhou 310016, China; MOE Key Laboratory of Macromolecular Synthesis and Functionalization, Department of Polymer Science and Engineering, Zhejiang University, Hangzhou 310027, China; Assisted Reproduction Unit, Department of Obstetrics and Gynecology, Sir Run Run Shaw Hospital, Zhejiang University School of Medicine, Hangzhou 310016, China; Key Laboratory of Reproductive Dysfunction Management of Zhejiang Province, Hangzhou 310016, China; Assisted Reproduction Unit, Department of Obstetrics and Gynecology, Sir Run Run Shaw Hospital, Zhejiang University School of Medicine, Hangzhou 310016, China; MOE Key Laboratory of Macromolecular Synthesis and Functionalization, Department of Polymer Science and Engineering, Zhejiang University, Hangzhou 310027, China; Assisted Reproduction Unit, Department of Obstetrics and Gynecology, Sir Run Run Shaw Hospital, Zhejiang University School of Medicine, Hangzhou 310016, China; Key Laboratory of Reproductive Dysfunction Management of Zhejiang Province, Hangzhou 310016, China; Assisted Reproduction Unit, Department of Obstetrics and Gynecology, Sir Run Run Shaw Hospital, Zhejiang University School of Medicine, Hangzhou 310016, China; Key Laboratory of Reproductive Dysfunction Management of Zhejiang Province, Hangzhou 310016, China; Assisted Reproduction Unit, Department of Obstetrics and Gynecology, Sir Run Run Shaw Hospital, Zhejiang University School of Medicine, Hangzhou 310016, China; Key Laboratory of Reproductive Dysfunction Management of Zhejiang Province, Hangzhou 310016, China; Assisted Reproduction Unit, Department of Obstetrics and Gynecology, Sir Run Run Shaw Hospital, Zhejiang University School of Medicine, Hangzhou 310016, China; Key Laboratory of Reproductive Dysfunction Management of Zhejiang Province, Hangzhou 310016, China; Department of Obstetrics and Gynaecology, School of Clinical Medicine, LKS Faculty of Medicine, The University of Hong Kong, Hong Kong SAR 999077, China; Shenzhen Key Laboratory of Fertility Regulation, The University of Hong Kong Shenzhen Hospital, Shenzhen 518000, China; Department of Obstetrics and Gynaecology, School of Clinical Medicine, LKS Faculty of Medicine, The University of Hong Kong, Hong Kong SAR 999077, China; Shenzhen Key Laboratory of Fertility Regulation, The University of Hong Kong Shenzhen Hospital, Shenzhen 518000, China; Department of Obstetrics and Gynaecology, School of Clinical Medicine, LKS Faculty of Medicine, The University of Hong Kong, Hong Kong SAR 999077, China; Shenzhen Key Laboratory of Fertility Regulation, The University of Hong Kong Shenzhen Hospital, Shenzhen 518000, China; Assisted Reproduction Unit, Department of Obstetrics and Gynecology, Sir Run Run Shaw Hospital, Zhejiang University School of Medicine, Hangzhou 310016, China; MOE Key Laboratory of Macromolecular Synthesis and Functionalization, Department of Polymer Science and Engineering, Zhejiang University, Hangzhou 310027, China; Assisted Reproduction Unit, Department of Obstetrics and Gynecology, Sir Run Run Shaw Hospital, Zhejiang University School of Medicine, Hangzhou 310016, China; Key Laboratory of Reproductive Dysfunction Management of Zhejiang Province, Hangzhou 310016, China

**Keywords:** adipose-derived mesenchymal stem cells, collagen scaffold, endometrium, fibrosis, regeneration

## Abstract

Severe endometrium damage causes pathological conditions such as thin endometrium and intrauterine adhesion, resulting in uterine factor infertility. Mesenchymal stem cell (MSC) therapy is a promising strategy in endometrial repair; yet, exogenous MSCs still raise concerns for safety and ethical issues. Human adipose-derived mesenchymal stem cells (ADMSCs) residing in adipose tissue have high translational potentials due to their autologous origin. To harness the high translation potentials of ADMSC in clinical endometrium regeneration, here we constructed an ADMSCs composited porous scaffold (CS/ADMSC) and evaluated its effectiveness on endometrial regeneration in a rat endometrium-injury model. We found that CS/ADMSC intrauterine implantation (i) promoted endometrial thickness and gland number, (ii) enhanced tissue angiogenesis, (iii) reduced fibrosis and (iv) restored fertility. We ascertained the pro-proliferation, pro-angiogenesis, immunomodulating and anti-fibrotic effects of CS/ADMSC *in vitro* and revealed that the CS/ADMSC influenced extracellular matrix composition and organization by a transcriptomic analysis. Our results demonstrated the effectiveness of CS/ADMSC for endometrial regeneration and provided solid proof for our future clinical study.

## Graphical Abstract

**Figure:**
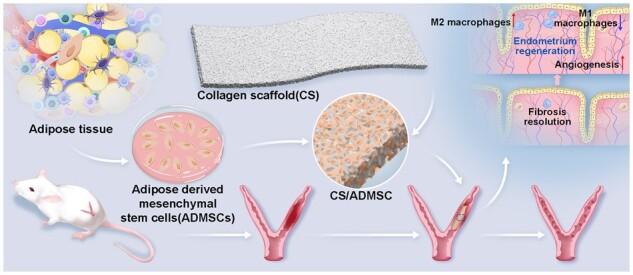

## Introduction

Endometrium plays an essential role in human reproduction. As one of the most dynamic tissues in the female body, it undergoes sequential proliferative phase, secretory phase and menstrual phase in a normal menstrual cycle under the endocrine environment. The menstruation and the following repair signify a typical natural injury and a scarless wound-healing process that occurs about 400 times during women’s reproductive time span [1]. Severe endometrium damage causes pathological conditions such as thin endometrium and intrauterine adhesion, resulting in uterine factor infertility [2, 3]. Therefore, promotion of endometrial regeneration can assist treatment of endometrium-related infertility. Conventional treatments including hysteroscopic adhesiolysis followed by hormone replacement can improve endometrial regeneration, but most patients with moderate to severe intrauterine adhesion still face a high risk of recurrence and poor pregnancy outcomes [4].

Mesenchymal stem cells (MSCs) are a group of adult stem cells found in many tissues including bone marrow, umbilical cord, placenta and adipose tissue. MSCs maintain a unique undifferentiated status and have multi-lineage differentiation potentials, indicating that they participate in tissue regeneration [5]. MSCs also display special paracrine profiles as they secrete a variety of trophic and immunomodulatory factors including growth factors, cytokines, chemokines and extracellular vesicles [6]. The trophic and immunomodulation effects of MSCs have been widely used in the treatment of degenerative diseases and tissue engineering [7, 8] such as bone repair [9, 10], skin repair [11, 12] and endometrial regeneration [13–16]. Most of the previous researches using MSCs in endometrium regeneration chose exogenous MSCs, which raised concerns for safety and accompanied ethical issues; therefore, autologous MSCs were drawing more attentions [17]. Adipose-derived MSCs (ADMSCs) reside in adipose tissue and exhibit better self-renewal ability compared with other MSC counterparts and could be easily and safely obtained by outpatient liposuction procedures [18]. The easy accessibility of ADMSCs makes it possible to achieve an autologous stem cell transplantation with the least ethical issues and the lowest immunogenic risks [19, 20]. Therefore, ADMSC is an ideal candidate for clinical translation.

Because of the anatomical feature of uterine cavity, intrauterine transplanted MSCs can easily be discharged out of the uterine cavity, thus reducing the local retention and effects of MSCs. In a murine model of endometrial injury, researchers observed only 0.045% of the transplanted MSCs engrafted into the endometrium 2 weeks after intrauterine injections [21]. The combined use of biomaterials and MSCs has been reported to enhance stem cell therapeutic effects [22–24]. Collagen is widely used and a naturally derived biomaterial with good biocompatibility and biodegradability [25, 26]. The porous structure of collagen-based scaffolds provides physical support for MSC adhesion and survival and enhances engraftment of MSCs at injury sites thus promoting tissue repair outcomes [27, 28]. Moreover, our previous clinical study using collagen-derived material proved the safety of collagen-based biomaterial for human use [14].

To exploit the regenerative effects of autologous ADMSCs and harness their high translational potential in future clinical use for endometrial regeneration, we evaluated the efficacy of ADMSCs on endometrial regeneration in a rat endometrium-injury model. Moreover, we constructed a collagen scaffold and loaded ADMSCs (CS/ADMSC) on it to deliver the stem cells into the uterine cavity. Histological analysis of the endometrium and fertility test revealed that the CS/ADMSC implantation significantly improved endometrial repair and restored fertility. *In vivo* and *in vitro* results validated the prominent anti-fibrotic and anti-inflammatory effects of the CS/ADMSC. Besides, our transcriptomic analysis revealed that the CS/ADMSC exerted its anti-fibrotic effects by influencing the components and organization of extracellular matrix (ECM).

## Materials and methods

### Culture and characterization of ADMSCs

ADMSCs were obtained from Re-Stem Biotech, Jiangsu, China. A third-party company (Kingmed Diagnostics, Guangzhou, China) confirmed the identity of the ADMSC using a flow cytometric method using a panel of recognized ADMSC surface markers, including positive expression for CD49d, CD73, CD90, CD105 and negative expression for HLA-DR, CD14, CD34, CD45. The ADMSCs were cultured in Dulbecco's Modified Eagle Medium/Ham’s F-12 (DMEM/F12) (Meilunbo, MA0214) supplemented with 10% fetal bovine serum (FBS) (Cellmax, SA101.02), 100 U/ml penicillin (Biosharp) and 100 mg/ml streptomycin (Biosharp) in an incubator (37°C, 5% CO_2_). The cells were trypsinized and passaged when they reached 90–100% confluence. Cells at Passages 4–6 were used for experimentation. The adipogenesis, osteogenesis and chondrogenesis abilities of ADMSCs were verified using an adipogenic differentiation kit (CYAGEN, HUXMD-90031), an osteogenic differentiation kit (CYAGEN, HUXMD-90021) and a chondrogenic differentiation kit (CYAGEN, HUXMD-90041) according to manufacturer’s protocols.

### Isolation and culture of primary human endometrial stromal cells

The use of human tissue or cells was approved by the ethics committee of Sir Run Run Shaw Hospital, Zhejiang University School of Medicine. Written informed consents were obtained from women aged 25–40 years old who underwent hysteroscopic examination of endometrium with biopsy 3–7 days after the completion of menstruation. Endometrial samples (about 0.3 g) were collected from those having regular menstrual cycles and no evidence of neither endometritis nor endometriosis. The tissue was rinsed with sterile PBS for 3 times, then cut into <1-mm^3^ pieces before dispersion in DMEM/F12 medium supplemented with collagenase III (0.5 mg/ml, Worthington LS004182) and DNase (0.2 mg/ml, Worthington LS002139) at 37°C in a shaking water bath for 60 min. Enzyme dispersion was terminated by adding an equal volume of DMEM/F12 with 10% FBS. The isolated endometrial stromal cells were obtained by using a 70-µm sieve to filter the undigested tissue pieces. The effluent was centrifuged at a speed of 800 rpm for 5 min. The cells were seeded onto a 10-cm dish. After 24 h, the culture medium (DMEM/F12 with 10% FBS, 100 U/ml penicillin and 100 mg/ml streptomycin) was changed and the adherent human endometrial stromal cells (HESCs) were further cultured till 90–100% confluence for passages. Cells from Passage 4 (P4)–P7 were used for experimentations.

### Preparation and characterization the ADMSC/collagen scaffold construct (CS/ADMSC)

The collagen scaffold was constructed by dehydrothermal treatment as previously reported [13]. Briefly, the collagen extracted from fresh bovine tendon was dissolved in acetic acid solution (3% w/v) at 37°C. The soluble extract was transferred into a Teflon mold, followed by lyophilization, then the lyophilized scaffold was crosslinked by vacuum dehydration at 105°C for 15 h. Additionally, the collagen scaffold was cut into uniform size (2.5 cm × 0.5 cm). CCK8 (Cell Counting Kit 8) assay and *in vitro* biodegradation experiment proved good cytocompatibility and biodegradability of CS (Supplementary Figs S1 and S2). To prepare CS/ADMSCs, 1 × 10^6^ cultured ADMSCs in 50 µl were seeded on a sterilized CS, and the composite was stabilized for 2 h in an incubator and then covered with culture medium for later use.

The morphologies and the microstructures of CS and CS/ADMSCs were observed by hematoxylin and eosin (HE) staining and by scanning electron microscope (SEM) (Hitachi S-4800, Japan). Intrauterine biodegradation was evaluated by HE staining the uterine horns (Supplementary Fig. S4).

### Growth and stemness of ADMSCs cultured on CS

Pieces of CS (0.5 cm × 05 cm) were placed into an ultra-low attachment 96-well plate (Corning 7007). A total of 5000 live ADMSCs in 200 µl of culture medium were seeded into the wells with or without CS. CCK8 assays were performed at different time points with three replicates in each group. The absorbance was measured under a 450-nm wavelength.

To evaluate the stemness of ADMSCs on CS, 1 × 10^6^ ADMSCs were seeded on the CS. The composite was stabilized for 2 h and then transferred into an ultra-low attachment 24-well plate (Corning 3473). After culture for 24 h, the composite was digested with collagenase III (0.5 mg/ml) in DMEM/F12 for 20 min. The cells were collected by filtration and centrifugation. An RNeasy micro kit (QIAGEN 74004) was used to extract RNA according to the manufacturer’s protocol. After reverse transcription, real-time qPCR was performed to detect the expression level of stemness-related genes Nanog, Sox2 and OCT4 (primers are listed in Supplementary Table S1). ADMSCs cultured on a normal attachment plate were used as a control.

### Effects of CS/ADMSCs on T-HESC cell proliferation

A total of 20 000 live T-HESCs (human telomerase reverse transcriptase-immortalized endometrial stromal cells, ATCC, CRL-4003) were seeded in each well of a 24-well plate, and CS/ADMSCs (0.25 cm × 0.25 cm with 2000 ADMSCs) were placed on the upper Transwell chamber (Labselect 14321). Blank upper chambers were used as control. On Day 0, Day 1 and Day 3, the upper chambers were removed for the CCK8 assays.

### Tube formation assay *in vitro*

To evaluate the angiogenetic effects of CS/ADMSC, we performed the tube formation assay. Human umbilical cord vein endothelial cells (HUVECs, Procell Ltd, HTX2104, Wuhan, China) were seeded onto 24-well plates precoated with Matrigel (Corning, 356231). CS/ADMSCs were placed in the upper Transwell chambers (Labselect 14321) and cocultured with HUVECs. Images were taken at 6 and 12 h after coculture (4 fields/well) and the extent of tube formation was analyzed using an ImageJ angiogenesis analyzer (https://imagej.nih.gov/ij/macros/toolsets/).

### Anti-fibrotic effects of CS/ADMSC *in vitro*

Using the CS/ADMSC and HESC coculture setup described above. Human recombinant TGFβ1 at a concentration of 10 ng/ml (PEPROTECH 100-21) was added to the culture medium to induce cellular fibrosis [29]. HESCs were divided into three groups: (i) cultured in normal medium; (ii) cultured with TGFβl; and (iii) cultured with TGFβ1 and CS/ADMSCs. The plates were placed in a 37°C incubator with 5% CO_2_ for 48 h.

For western blotting, the lysate of HESCs was added into the Bio-Rad loading buffer (BioRad 1610747) according to the manufacturer’s instruction. Protein solutions were run on 10% SDS-PAGE gel and transferred to PVDF membrane. The membranes were blocked in 5% skim milk before incubation with the following primary antibodies at 4°C overnight: antibodies against collagen I (COLA1, 1:1000, Abcam ab138492), fibronectin (FN, 1:1000, Sigma-Aldrich, F3648), α-smooth muscle actin (αSMA, 1:10 000, Abcam, ab124964), phosphorated Smad3 (pSmad3, 1:1000, CST, 9520S) and glyceraldehyde-phosphate dehydrogenase (GAPDH, 1:5000, Proteintech, 10494-1-AP). The membranes were then incubated with HRP-conjugated secondary antibodies (1:5000; BD Pharmingen, 554021 and 554022) at room temperature for 1 h. An enhanced chemiluminescence reagent (Millipore) was added to the membrane and the signal was immediately detected by a BioRad ChemiDoc. GAPDH was used as the internal reference.

For RT-QPCR detection, the RNA of HESC was extracted using an RNA extraction kit (ES Science RN001) and the manufacturer’s protocol. Primers of target genes are listed in Supplementary Table S1. The expression of each gene was standardized using GAPDH expression as a reference.

### Transcriptomic analysis of CS/ADMSC’s effects on HESCs

HESCs were prepared as described in the ‘Anti-fibrotic effects of CS/ADMSC *in vitro*’. For this study, HESCs were divided into three groups in triplicate and were labeled as: CTRL (cultured in normal medium), T (cultured with TGFβ1) and T/C.A (cultured with TGFβ1 and CS/ADMSCs). TRIzol reagent was used to lyse the HESCs and samples were preserved in −80°C refrigerator before experimentation. RNA-sequencing was conducted using an Illumina NovaseqTM6000 (LC Bio Technology Co., Ltd. Hangzhou, China). To evaluate gene expression levels of HESCs in different groups, reference genome alignment was performed using Hisat2, and Stringtie was used to reconstruct transcripts and calculate the expression levels of all genes in each sample. Fragments per kilobase million values were used to represent and to compare the gene expression levels in different samples. In differential gene expression analysis, DESeq2 and edgeR methods were applied to identify significant reads out differences, and BH-adjusted *P* (*P*.adj) values <0.05 were considered significant for the analyses. Gene ontology (GO) analysis was performed to evaluate the enriched biological terms and processes in different groups.

### Immunomodulatory effects of CS/ADMSC *in vitro*

We used murine abdominal macrophages to determine whether CS/ADMSCs exerted immunomodulatory effects. To isolate murine abdominal cells, 2 ml of 4% thioglycolate broth was intraperitonially injected into female C57BL/6 mice 72 h before the mice were sacrificed. 10 ml of sterile PBS was intraperitoneally injected to flush out the macrophages. The cells were cultivated in RPMI-1640 medium supplemented with 10% FBS with or without CS/ADMSCs coculture for 48 h. Lipopolysaccharide (LPS) (Invitrogen, USA) (100 ng/ml) was added 6 h before RNA extraction or flow cytometry analysis.

The primers used for RT-QPCR are listed in Supplementary Table S1. For flow cytometry tests, the cells were stained with FITC-conjugated anti-F4/80 antibody (1:100, Invitrogen 11480182), PE-conjugated anti-CD86 antibody (1: 160, Invitrogen 12086282) and APC-conjugated anti-CD163 antibody (1: 80, Invitrogen 17163182) for 30 min and then detected using a Beckman DXFLEX flow cytometer.

### Rat model of acute endometrial injury and implantation

The animal experiment was approved by the animal ethics committee, Zhejiang University, and the ethics committee of Sir Run Run Shaw Hospital, Zhejiang University School of Medicine. Female Sprague-Dawley (SD) rats of 8–10 weeks old (220–250 g) were purchased from the Experimental Animal Center of Zhejiang Province, China, and were raised in a specific pathogen-free environment with a day/night cycle of 12/12 h at a room temperature of 22–25°C. The animals were allowed free access to water and food.

The experimental design is shown in Fig. 2A. Briefly, pregnant mere serum gonadotropin was administrated intraperitonially at a dosage of 200 U/kg to synchronize the animals’ uterine cycles 48 h before surgery. An acute endometrial injury model was established as previously described with a few modifications [15]. Rats were anesthetized with 2% phenobarbital sodium (50 mg/kg) intraperitonially. A 3-cm longitudinal excision was made at the midline of the lower abdominal area to expose the uterine horns. A small incision of 0.2 cm was made at the cervical junction site of the right horn and a hemostatic clap was placed at the distal end of the horn to protect the oviduct opening. A 1-ml syringe was used to inject ∼200 µl of 95% ethanol into the horn through the cervical junction cut, which was then clamped for 2 min before flushing the ethanol out with PBS (20 ml). To implant the CS or CS/ADMSC, a pair of thin tweezers were used to insert and spread the CS or CS/ADMSC into the uterine horn through the incision and the wound opening was stitched with 6-0 absorbable suture. The left horn remained intact as the normal control. Benzylpenicillin potassium was then given intraperitonially for 3 days for prophylaxis. The rats were randomly divided into three groups: natural repair (NR) group (damage without treatments), CS group (damage and CS implantation) and CS/ADMSC group (damage and CS/ADMSC implantation).

### Histological staining and immunohistochemistry

On postoperation Day 14 and Day 28, the rats were sacrificed, and the treated horns were fixed in 4% paraformaldehyde (PFA) solution, dehydrated and embedded in paraffin. The paraffin-embedded samples were sliced into 7-µm sections for staining.

HE and Masson trichrome staining were performed with standard protocols. Images of 4 high-power fields (HPF) for each slide were captured using an optical microscope (ZEISS, Scope.A1). The endometrial thickness and the proportion of fibrotic area were analyzed by the FIJI software (https://fiji.sc/).

For immunohistochemistry (IHC) staining, the slides were subjected to routine deparaffination, rehydration and antigen retrieval at 100°C in 10 mM citrate sodium buffer. Endogenous peroxidase activity was blocked with 3% H_2_O_2_ and, then, the slides were blocked in 5% bovine serum albumin (BSA) for 1 h at room temperature. The slides were incubated with primary antibodies against Ki67 (1:200, ab15580, Abcam) and CD31(1:50, ab281583, Abcam) in 5% BSA at 4°C overnight and then rinsed and subjected to incubation with HRP-conjugated secondary antibody for 1 h before being exposed to a DAB solution. The slides were observed under an optical microscope and 4 HPF on each slide were captured for analysis.

### Immunofluorescence

Female SD rats were divided into three groups as described above. On Day 3 and Day 7 postoperation, the rats were sacrificed, and the right uterine horns were collected and fixed in 4% PFA solution. After dehydration and paraffin embedding, the samples were sliced into 7-µm sections for immunofluorescence staining. The slides were subjected to deparaffination, rehydration and antigen retrieval and washed with PBS for three times. They were then blocked with 5% BSA for 1 h at room temperature. Subsequently, primary antibodies diluted in 5% BSA were added on the slides and incubated overnight at 4°C. The slides were rinsed with PBS 3 times, and then, DAPI was added to stain the nuclei. The primary antibodies used were anti-F4/80 antibody (1:500, Abcam, ab300422), PE-conjugated anti-CD86 antibody (1:50, Biolegend, 200307) and anti-CD163 antibody (1:200, Abcam, ab182422). The slides were observed under a laser confocal fluorescence microscopy (ZEISS, LSM800). Images were captured under uniform laser excitation and imaging conditions.

### Fertility assessment

A total of 40 female rats (including non-pregnant ones) were used for fertility assessment. The rats were assigned into three groups and treated as described above. On Day 28 post-surgery, female rats were allowed to mate with male rats at a F/M ratio of 2/1. On Day 18 after mating, the female rats were sacrificed, and the uterine horns were exposed. The presence of embryos in the left and/or the right horns indicated successful pregnancy and the rats were included in the fertility test analysis. Right horn pregnancy rate and relative embryo implantation ratio compared to the left horn were calculated.

### Statistical analysis

Experiment data were presented in mean ± standard deviation. One-way analysis of variance tests with multiple comparisons were performed to determine the significance among groups when the data fit normal distribution. The Kruskal–Wallis test was used to compare groups with non-normal distribution of results. Pregnancy rates and relative embryo implantation ratios were compared using Chi-square test or Fisher’s exact test where appropriate. Statistical differences were considered significant when *P* < 0.05.

## Results

### Multilineage differentiation and surface marker expression of ADMSCs

ADMSCs express surface markers of typical MSCs as well as unique surface signatures [30]. Flow cytometry verified that almost all the isolated ADMSCs expressed CD49d (99.69%), CD73 (100%), CD90 (99.97%) and CD105 (100%) but with minimal expression of HLA-DR (0.02%), CD14 (0.01%), CD34 (0.16%) and CD45 (0.09%) (Supplementary Fig. S3). Multi-lineage differentiation experiments demonstrated that the ADMSCs could undergo osteogenesis, chondrogenesis and adipogenesis when cultured in the respective differentiating media (Supplementary Fig. S3). The results demonstrated that the ADMSCs fulfilled the criteria of The International Society for Cellular Therapy suitable for stem cell therapy [31].

### Morphological characterization of CS and CS/ADMSC

**Figure 1.**
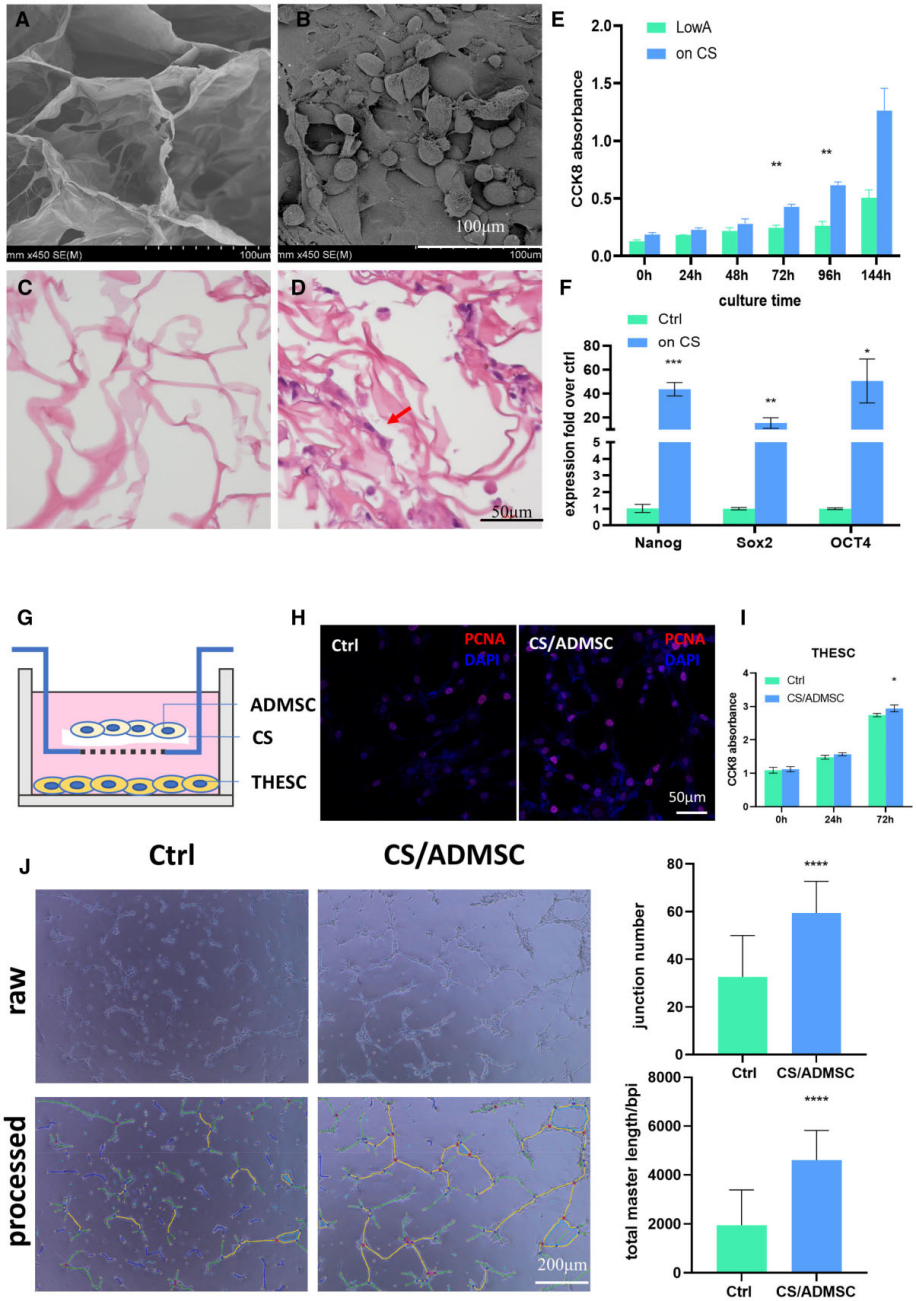
Morphological and functional characterization of CS and CS/ADMSC. (**A**, **B**) SEM images of CS and CS/ADMSC, red arrow indicating ADMSCs on CS. (**C**, **D**) HE staining of CS and CS/ADMSC. (**E**) CCK8 absorbance indicated ADMSC’s growth and proliferation on CS (LowA: low attachment plate). (**F**) ADMSC cultured on CS exhibited higher stemness compared with control. (**G**) A schema of the transwell coculture system. (**H**, **I**) CS/ADMSC promoted T-HESC proliferation. (**J**) Tube formation assay proved the pro-angiogenetic effect of CS/ADMSC. *P < 0.05, **P < 0.01, ***P < 0.001, ****P < 0.0001.

The CS showed a porous structure under HE staining and SEM with a mean pore size of ∼110 µm. (Fig. 1A and C). When ADMSCs were loaded onto the CS, the porous structure provided essential sites for the cells to adhere and to grow to spindle shape as those on culture plates (Fig. 1B and D).

### Cell growth and stemness of ADMSCSs on CS

Adherent cells need to attach to a solid surface to grow and proliferate *in vitro*. Therefore, we evaluated whether CS could provide the physical support for ADMSCs. CCK8 assays indicated that the ADMSCs exhibited poor growth when cultured on ultra-low attachment plates (Fig. 1E). However, the ADMSCs showed remarkable proliferation in the presence of CS as indicated by the ascending CCK8 absorbance after 72 and 96 h of culture (Fig. 1E). Moreover, the ADMSCs cultured on CS expressed higher levels of stemness-related genes (Fig. 1F) compared to those cultured on conventional plates. These results indicated that CS could be an ideal vector for stem cell transplantation *in vivo* because it supported the growth and retained stemness of ADMSCs.

### Effects of CS/ADMSC on endometrium-related cells *in vitro*

MSCs promote cell growth by their paracrine actions. A coculture system was used to evaluate whether CS/ADMSC promoted endometrial stromal cell growth (Fig. 1G). T-HESCs showed ascending CCK8 absorbance when cultured in the presence or absence of CS/ADMSC (Fig. 1I). On Day 0 and Day 1, the absorbance values in the two groups were similar. On Day 3, the T-HESCs in coculture with CS/ADMSC showed significantly higher absorbance compared to the control group. The expression of proliferating cell nucleus antigen (PCNA) was also higher in the cocultured T-HESCs (Fig. 1H). The results proved that the CS/ADMSC promoted endometrial cell growth. The result of *in vitro* tube formation assay also indicated that the CS/ADMSC increased the tube formation abilities of HUVEC; the total branch length and junction number of HUVEC were both higher in the presence of CS/ADMSC (Fig. 1J).

### HE staining and Ki67 IHC staining

In normal menstrual cycles, endometrium undergoes proliferation under the influence of rising serum estradiol levels to reach a certain thickness before transforming into the secretory phase. Although rodents do not have a typical menstrual phase, their endometrium thickens in a similar pattern [32]. Adequate proliferation is an important endometrial feature to ensure the retention of substantial tissue after menstruation for the subsequent cycle. Therefore, proliferation is an important process in endometrial regeneration.

**Figure 2.**
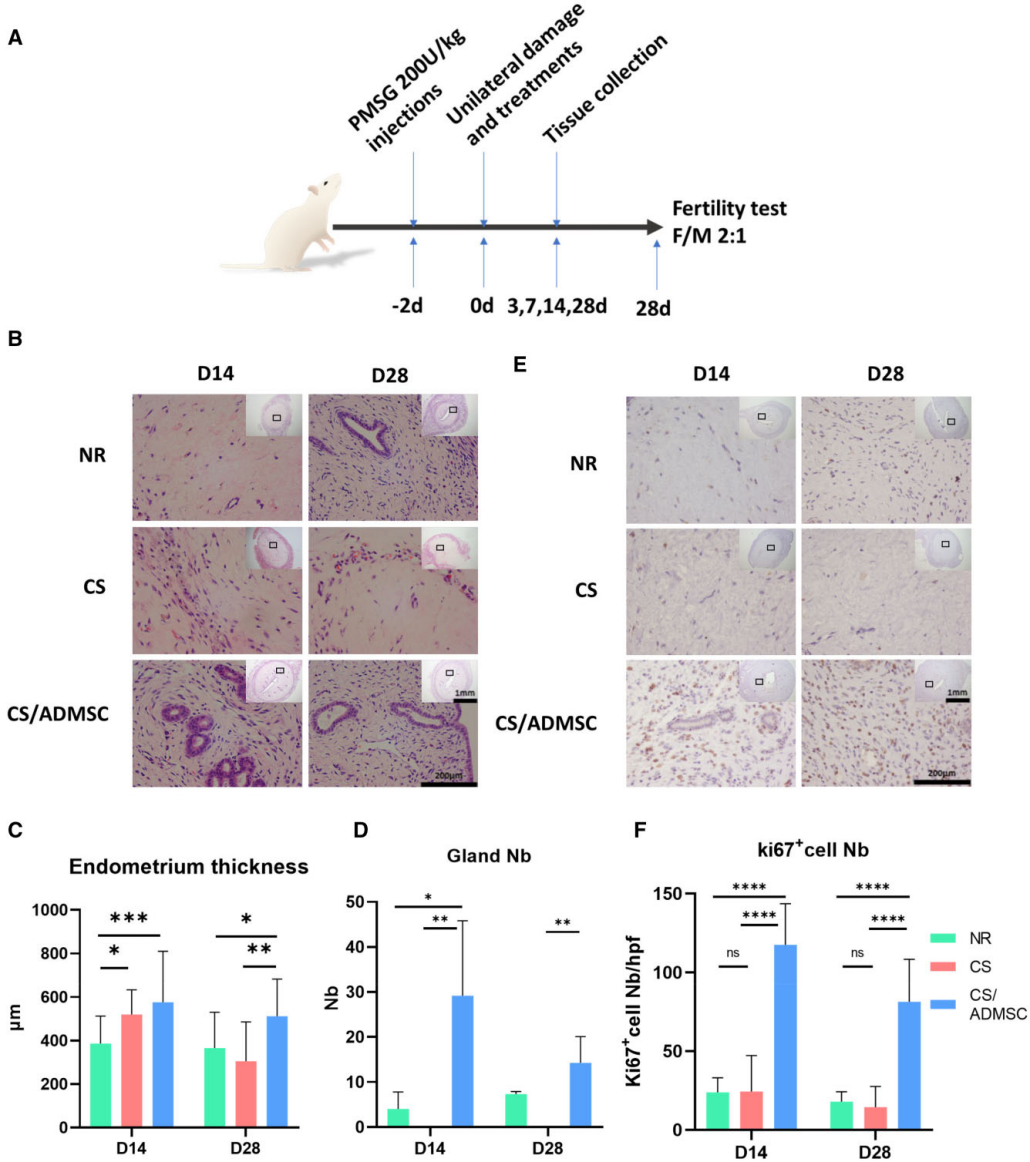
CS/ADMSC Promoted endometrial growth after acute damage. (**A**) Animal experiment design schema. (**B**) HE staining of uteri of the groups. Statistical analysis of (**C**) endometrial thickness and (**D**) gland numbers among groups. (**E**) Ki67 immunohistochemistry of uteri among the 3 groups. (**F**) Statistical analysis of the number of ki67-positive cells under high-power fields. *N* ≥ 3, 4 high-power fields were captured on each section. *P < 0.05, **P < 0.01, ****P < 0.0001.

To understand how CS/ADMSC help in preserving endometrial functions after induced injury, we compared the morphology of the endometrium of rat in each experimental group shown in Fig. 2A. On Day 14, the endometrial thickness of the CS/ADMSC group and the CS group were significantly higher than that of the NR group. The higher endometrial thickness of the CS/ADMSC group continued till Day 28 (*P* < 0.05 vs the NR group, *P* < 0.01 vs the CS group), while the overall size of the endometrium was reduced remarkably in the CS group on Day 28 (Fig. 2B and C). Besides endometrial thickness, endometrial glands are also responsible for endometrial functions and therefore, their number can also indicate endometrial regeneration outcomes. As shown in Fig. 2B and D, the gland numbers of the CS/ADMSC group were significantly higher than those of the NR and the CS group (0 glands observed under microscopy) on both Day 14 and Day 28. Ki67 is a protein involved in mitosis and is a marker for cell proliferation [33]. The number of ki67 positive cells was high in the CS/ADMSC group (Fig. 2E and F), indicating active proliferation in the endometrium. However, the ki67 positive cells were sparse in the NR and CS groups (Fig. 2E and F). The result showed that the CS/ADMSC retained the proliferative capacity and promoted the regeneration outcomes of the injured endometrium.

### Effects of CS/ADMSC on endometrial angiogenesis and fibrosis

Intact blood vessel networks are required for nutrients and oxygen supply, as well as waste removal. Therefore, the reconstruction of blood vessel networks is a pivotal step for successful tissue repair. CD31 is highly expressed in vascular endothelial cells and is commonly regarded as an indication of active angiogenesis [34].

**Figure 3.**
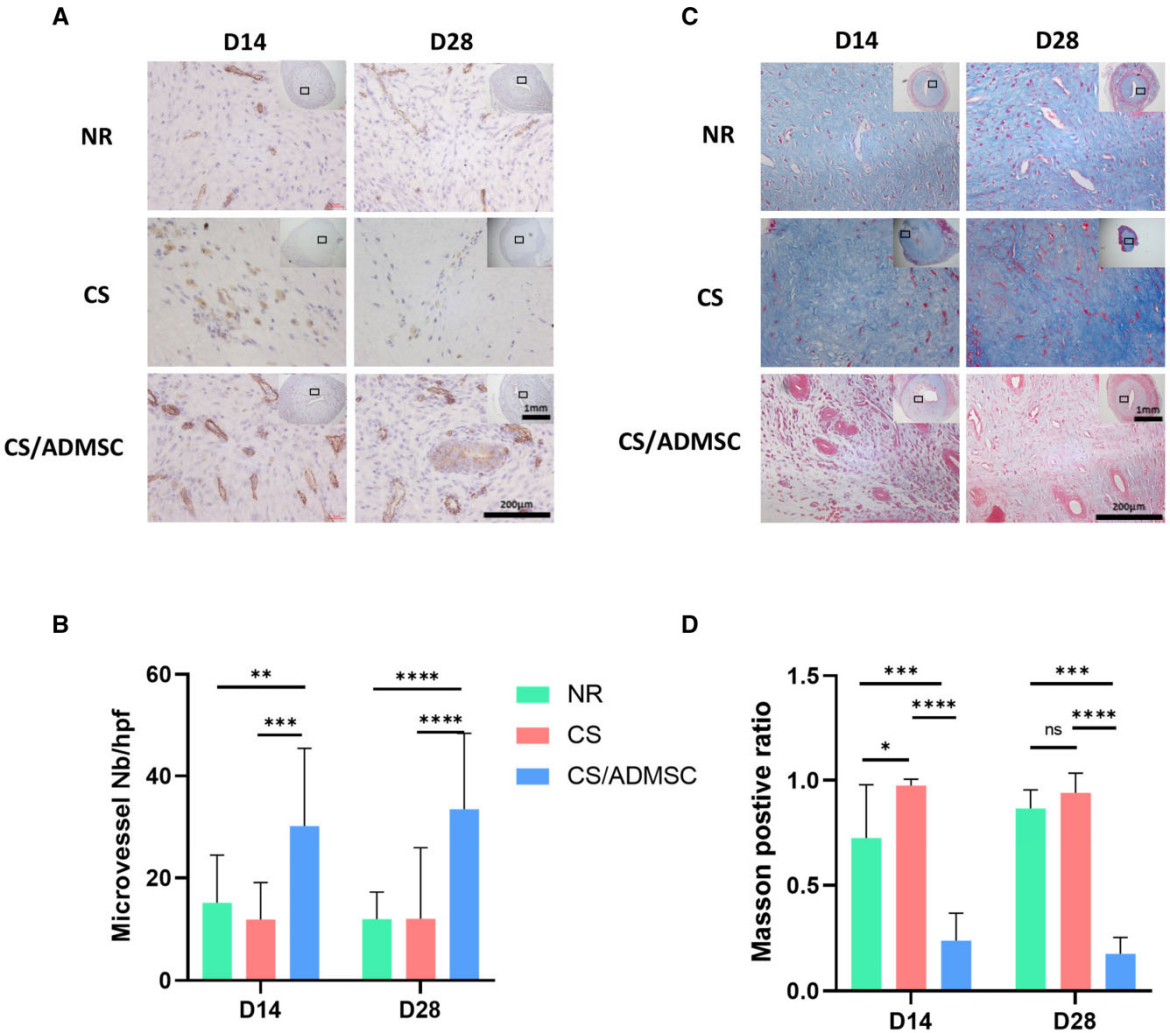
CS/ADMSC implantation promoted angiogenesis and ameliorated fibrosis *in vivo*. (**A**) CD31 immunohistochemistry of uteri. (**B**) Quantification and statistical analysis of CD31-positive microvessels among groups. (**C**) Masson trichome staining of uteri. (**D**) Semi-quantification and statistical analysis of the positive ratio of blue-stained area on each image. *N* > 3, 4 high power fields were captured on each slide. *P < 0.05, **P < 0.01, ***P < 0.001, ****P < 0.0001.

Based on CD31 IHC, the endometrium of the CS/ADMSC group showed a higher newly formed microvessel density than the NR and CS groups on both Day 14 and Day 28 (Fig. 3A and B), demonstrating that the CS/ADMSCs implantation enhanced the angiogenesis of the endometrium post-damage.

Abnormal deposition of excessive ECM characterizes one of the pathological features of intrauterine adhesion. The transformation of stromal fibroblasts to myofibroblasts severely sabotaged the normal function of endometrial stromal cells (fibroblasts). Moreover, the deposition of ECM would stiffen the tissue, leading to the replacement of normal endometrial tissue by dysfunctional fibrotic tissue. Therefore, preventing tissue fibrosis post-trauma is crucial for functional tissue regeneration.

To evaluate whether CS/ADMSCs could reduce ECM deposition and ameliorate endometrial fibrosis, Masson trichrome staining was performed on tissue samples collected on Day 14 and Day 28 post-surgery and the collagen-positive area (in blue) was semi-quantified (Fig. 3C and D). On Day 14, both the NR and CS groups showed severe tissue fibrosis with fibrosis ratios of 0.72 ± 0.26 and 0.97 ± 0.03, respectively. In the CS/ADMSC group, the fibrosis ratio was 0.24 ± 0.13, which was significantly lower than that of the NR group (*P* < 0.001) and the CS group (*P* < 0.0001). Similar outcomes were observed on Day 28; the fibrosis ratio of the CS/ADMSC (0.18 ± 0.08) was lower than that of the NR group (0.86 ± 0.09 *P* < 0.001) and the CS group (0.94 ± 0.09 *P* < 0.0001). These results indicated that the CS/ADMSC reduced abnormal ECM deposition and prevented endometrial fibrosis. We also noticed that the fibrosis was more pronounced in the CS group than the NR group. This phenomenon could be explained by the foreign body reaction of host tissue to solid implants. Uncontrolled foreign body reaction could result in lasting inflammation, excessive fibrosis and failure of tissue repair [35].

### Immunomodulation effects of CS/ADMSC *in vitro* and *in vivo*

After tissue injury, the immune system initiates an acute inflammatory response to clear tissue debris and to fight against pathogens [36]. Macrophages are recruited to the injured sites soon after neutrophils to participate in the pro-inflammatory phase, mainly to clear debris by efferocytosis. Prolonged inflammation not only sabotages regeneration but also leads to tissue fibrosis [37]. A timely resolution of inflammation and phenotype switch of macrophages from pro-inflammatory to pro-regenerative are indispensable for following tissue regeneration [37–40].

**Figure 4.**
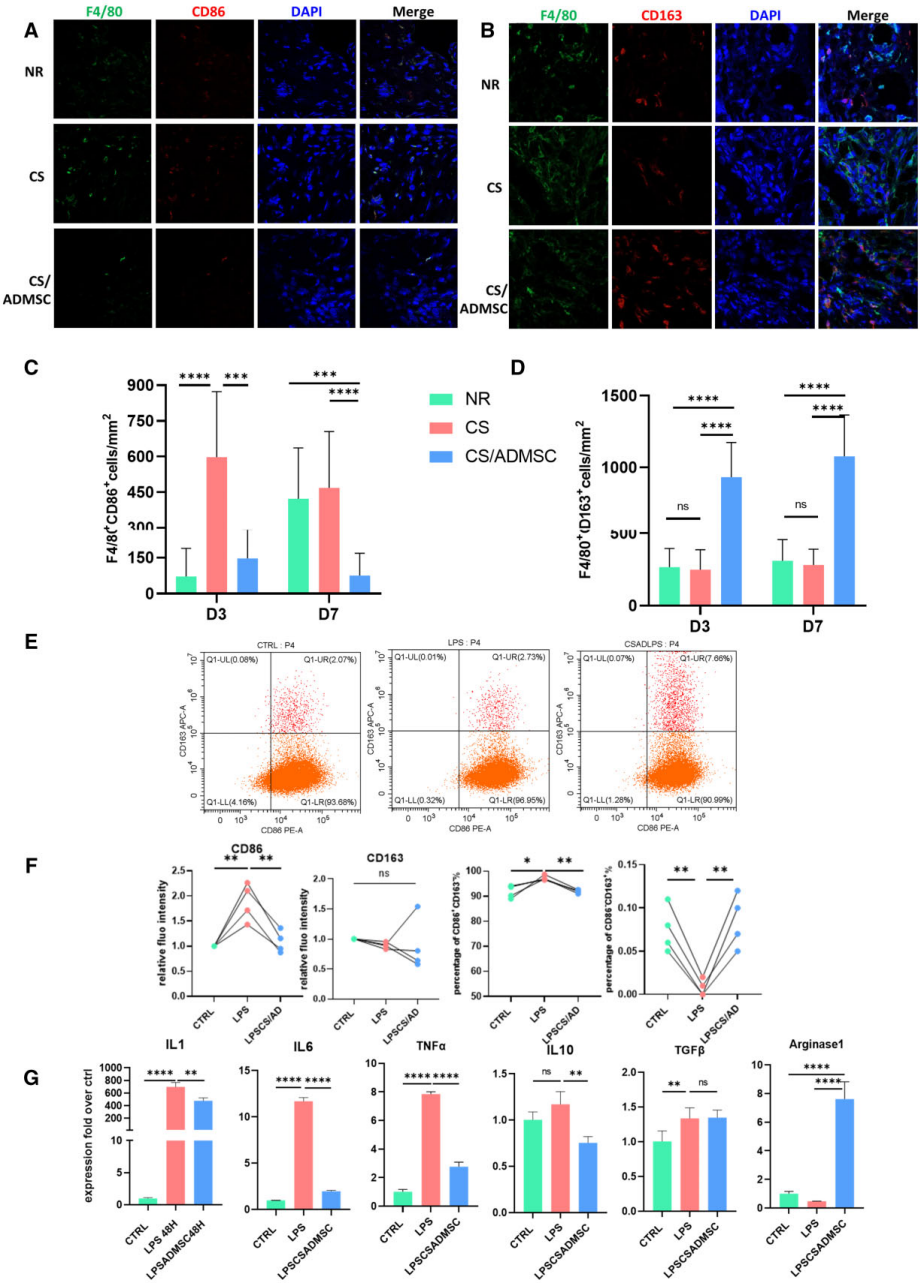
The immunomodulation effects of CS/ADMSC *in vivo* and *in vitro*. Immunofluorescence staining of (**A**) pro-inflammatory (CD86+) macrophages and (**B**) anti-inflammatory macrophages (CD163^+^) in endometrium on Day 7, *N* ≥ 3. (**C** and **D**) Statistical results of the number of pro-inflammatory and anti-inflammatory macrophages. (**E**) Flow cytometry results of surface markers of macrophages with different treatments. (**F**) Statistical analysis of CD86 and CD163 relative fluorescence intensity, the proportion of CD86 single positive and CD163 single positive macrophages with different treatments. (**G**) Gene expression levels of macrophages in different groups. *P < 0.05, **P < 0.01, ***P < 0.001, ****P < 0.0001.

We evaluated the macrophage phenotypes *in situ* by immunofluorescence staining. On Day 3 post-operation, F4/80^+^ macrophages were observed in all three groups (Supplementary Fig. S5), indicating recruitment of macrophages to the damaged site. However, we observed a significant difference of the macrophage phenotypes among the three groups. On Day 3, the pro-inflammatory CD86^+^ macrophages were observed in all groups. They were the most prominent macrophages in the CS group (*P* < 0.0001 vs the NR group, *P* < 0.001 vs the CS/ADMSC group), and this prevalence of pro-inflammatory macrophages in the CS group continued till Day 7 when the pro-inflammatory macrophages in the NR group began to accumulate (Fig. 4A and C). The CS/ADMSC group showed significantly lower levels of the pro-inflammatory macrophages compared with the other groups (Fig. 4A and C). However, the anti-inflammatory and pro-regenerative CD163^+^ macrophages prevailed in the CS/ADMSC group on Day 3 (*P* < 0.0001) and Day 7 (*P* < 0.0001) compared with the NR and the CS group (Fig. 4B and D). The *in vivo* results showed that the CS/ADMSC implantation inhibited polarization of the pro-inflammatory macrophages, but promoted that of the pro-regenerative macrophages. The anti-inflammatory effects of CS/ADMSC were validated by flow cytometry and RT-QPCR. LPS-induced increase of the CD86^+^CD163^−^ macrophage proportion was suppressed by CS/ADMSC preconditioning (Fig. 4E and F), and the mean fluorescence intensity was also reduced in the CS/ADMSC preconditioned macrophages. Though the fluorescence intensity of CD163 showed no statistical significance, the proportion of CD163^+^CD86^−^ macrophages was remarkably higher in the CS/ADMSC preconditioned macrophages than that of the non-preconditioned macrophages after LPS stimulation (Fig. 4E and F). Macrophages also modulate the surrounding cells and inflammatory microenvironment by secreting cytokines. Pro-inflammatory cytokines, including IL1B, IL6 and TNFα, were remarkably downregulated, and the anti-inflammatory marker Arginase 1 was more highly expressed in the CS/ADMSC preconditioned macrophages than the non-preconditioned macrophages. These results demonstrate that CS/ADMSC exerted anti-inflammatory and macrophage modulatory effects *in vitro* and *in vivo*.

### Effects of CS/ADMSC on fibrotic marker expression of HESCs

**Figure 5.**
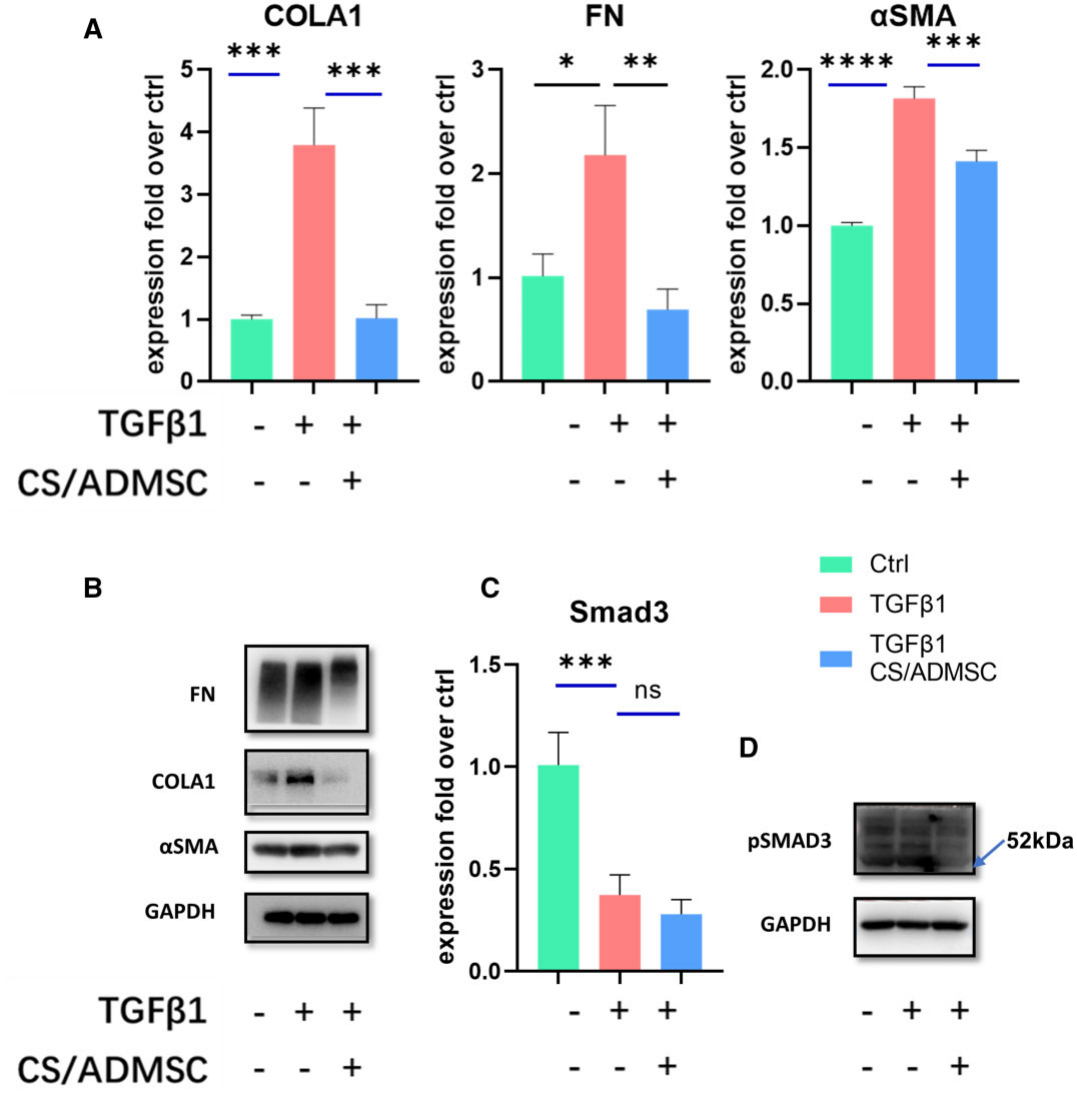
Anti-fibrotic effects of CS/ADMSC on primary HESCs. (**A**) Real-time qPCR analysis of the expression levels of fibrotic genes collagen 1 (COLA1), fibronectin (FN) and myofibroblast transformation marker α-smooth muscle actin (αSMA). (**B**) Western blotting detection of COLA1, FN and αSMA. (**C**) Real-time qPCR analysis of Smad3 expression levels. (**D**) Western blotting detection of phosphorylation levels of Smad3. *P < 0.05, **P < 0.01, ***P < 0.001, ****P < 0.0001.

TGFβ1 expression is correlated with the pathogenesis of intrauterine adhesion and other fibrotic diseases [41, 42]. When stimulated by TGFβ1, HESCs manifest a fibrotic phenotype similar to intrauterine adhesion [29]. We established a cell model of endometrial fibrosis by conditioning HESCs with rcTGFβ1. Increased expressions of fibrotic markers of collagen I and fibronectin were verified by RT-QPCR (Fig. 5A) and western blotting (Fig. 5B) when the HESCs were exposed to rcTGFβ1 only. However, when cocultured with CS/ADMSC, the expression of these fibrotic markers was reversed (Fig. 5A and B). αSMA is expressed in activated myofibroblasts, which are the culprit of ECM deposition in tissue fibrosis [43]. In HESCs, αSMA was upregulated by TGFβ1, indicating a fibroblast-to-myofibroblast transformation (Fig. 5A and B). This effect was restrained by coculture with CS/ADMSC (Fig. 5A and B), implying that the CS/ADMSC could inhibit the TGFβ1 induced fibroblast-to-myofibroblast transformation. Smad3 serves as a signal transduction molecule in the canonical TGFβ signal pathway, and the phosphorylated smad3 forms a heterotrimer complex that is translocated to nucleus for activation of the fibrosis-related gene expression [44]. We observed lower smad3 phosphorylation levels in the control HESCs and the CS/ADMSC cocultured HESCs than the HESCs upon exposure to TGFβ1 (Fig. 5C and D). This result illustrated that CS/ADMSC exerted anti-fibrotic effects through suppression of smad3 phosphorylation.

### Transcriptome-sequencing analysis

**Figure 6.**
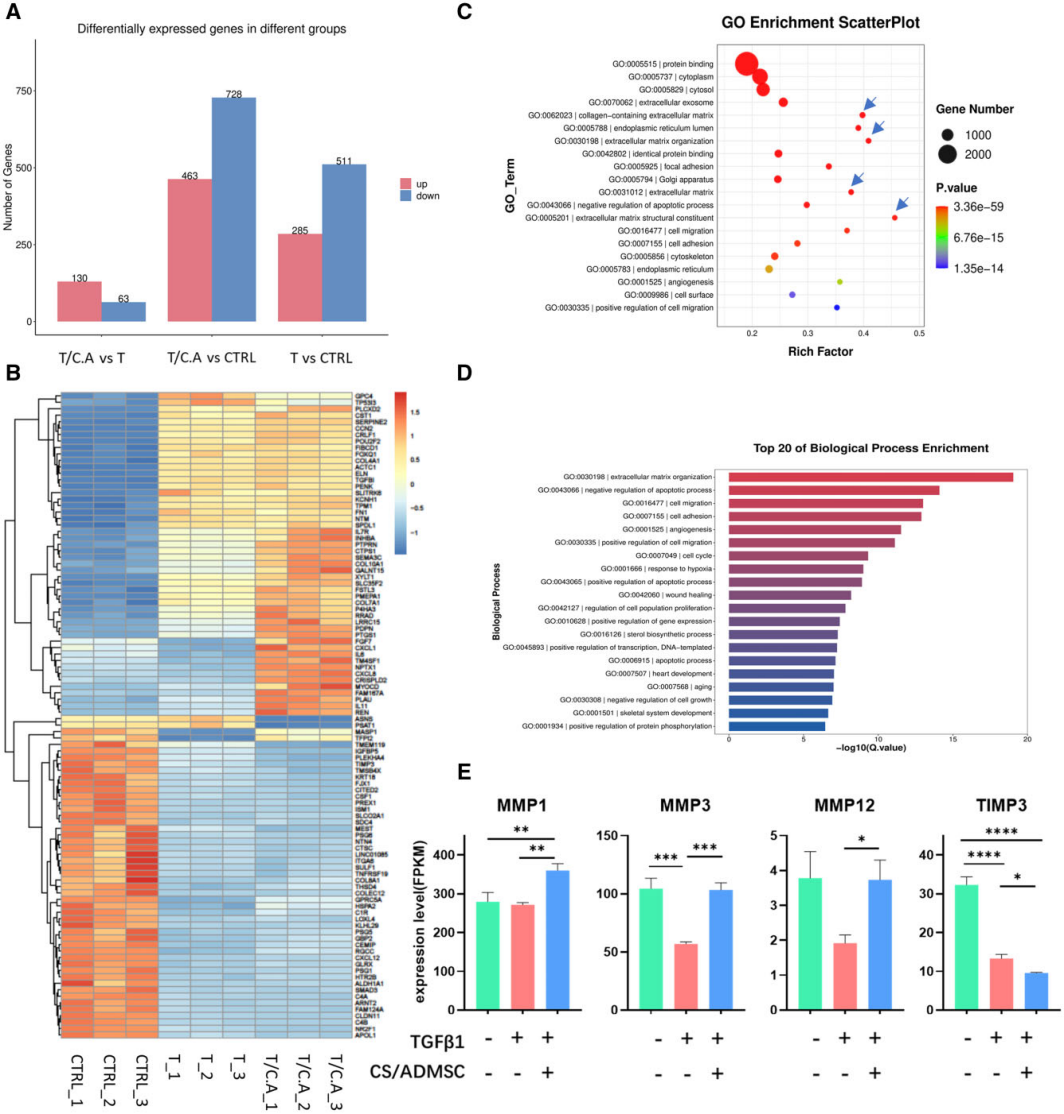
CS/ADMSC influenced ECM components and organization. (**A**) Gene differential expression in different groups. (**B**) Heatmap of top 100 differentially expressed genes in different groups. (**C**) ECM-related GO terms were remarkably enriched (blue arrows). (**D**) GO analysis identified ‘extracellular matrix organization’ as the top enriched biological process. (**E**) Differential expression levels of MMPs and TIMP3. *P < 0.05, **P < 0.01, ***P < 0.001, ****P < 0.0001.

To gain a deeper insight into the regenerative effects of CS/ADMSC, we applied mRNA transcriptome sequencing. Gene differential expression analysis identified 193 differentially expressed genes (130 up, 63 down) between the T/C.A and the T groups; 1191 differentially expressed genes (463 up, 728 down) between the T/C.A and the CTRL groups, 796 differentially expressed genes (285 up, 511 down) between the T and the CTRL group (Fig. 6A). The top 100 differentially expressed genes are shown in the heatmap (Fig. 6B). GO analysis revealed that 4 out of the top 5 enriched GO terms were related to ECM components and organization (Fig. 6C), namely GO:0005201|extracellular matrix structural constituent, GO:0030198|extracellular matrix organization, GO:0062023|collagen-containing extracellular matrix and GO:0031012|extracellular matrix. The top enriched biological process was ‘extracellular matrix organization’ (Fig. 6D). These genes were investigated and found to be highly related to the matrix metalloproteinase (MMP) family. MMPs are a family of proteinases that participate in ECM degeneration. Our observations suggested that the CS/ADMSCs had significant influences on ECM formation and organization, thus exerting anti-fibrotic effects to surrounding tissue when transplanted *in vivo*. MMPs and tissue inhibitors of MMPs (TIMPs) are involved in the pathogenesis of fibrosis [45]. Though MMPs show contradicting contributions to fibrosis progression or resolutions [46], the differentiated expression of MMPs in HESCs indicates that the CS/ADMSC may exert its ECM modulatory functions via modulating MMPs (Fig. 6E).

We also noted that the biological processes of GO:0042060|wound healing, GO:0007049|cell cycle, GO:0042127|regulation of cell population proliferation and GO:0043066|negative regulation of cell apoptosis were also remarkably enriched, consistent with the endometrial cell growth and proliferation effect of CS/ADMSC shown in Figs 1H and I and 2. The biological process GO:0001525|angiogenesis was also notably enriched (Fig. 6C). Other enriched cell processes involved in cell migration and cell adhesion might also played a role in endometrial repair (Fig. 6C and D).

### CS/ADMSC implantation restored fertility in rats

**Figure 7.**
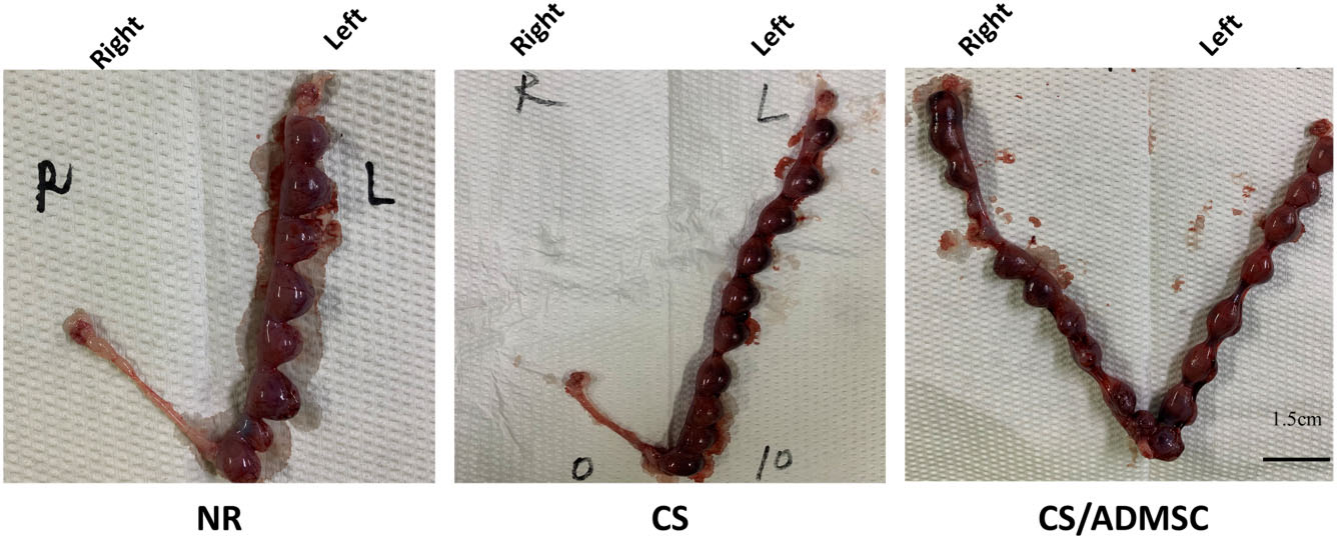
CS/ADMSC restored fertility: gross pictures showing the pregnancy outcomes of different groups.

On the 18th day after mating, female rats were sacrificed and their uteri were exposed. Representative images of the uteri from each group are displayed in Fig. 7. The pregnancy rate and relative embryo implantation ratio of the right uterine horn are summarized in Table 1. In the NR group, only 1 out of 11 rats had implantation sites in the right uterine horn. In the CS group, no implantation site was observed in right uterine horns. In the CS/ADMSC group, 50.00% of the rat had implantation site (*P* < 0.05 vs NR; *P* < 0.01 vs CS) and the relative embryo implantation ratio was 28.4% (*P* < 0.01 vs NR; *P* < 0.0001 vs CS). The results indicated that the CS/ADMSC implantation facilitated restoration of endometrial function and fertility after acute endometrial damage.

**Table 1.** Summary table of pregnancy rate and embryo implantation ratio of different groups

Groups	Total pregnant animal number	Right uteri pregnant animal number	Right uteri pregnant percentage (%)	Number of implantation sites in left uteri	Number of implantation sites in right uteri	Relative percentage of embryo implantation (%)
NR	11	1	9.1	81	6	7.4
CS	11	0	0	76	0	0
CS/ADMSC	14	7	50.0^a^^,^^b^	102	29	28.4^c^^,^^d^

a *P* < 0.05 CS/ADMSC vs NR.

b *P* < 0.01 CS/ADSMC vs CS.

c *P* < 0.01 CS/ADMSC vs NR.

d *P* < 0.0001CS/ADSMC vs CS.

## Discussion

Healthy endometrium possesses remarkable regenerative capacity and is an ideal model for studying the natural tissue repair process [47, 48]. However, endometrium can suffer severe damage as a result of surgical abortion, intrauterine procedures or infection [3]. The basal layer of endometrium is responsible for post-menstruation or postpartum endometrial regeneration. When the basal layer is damaged, the endometrium fails to regenerate, causing pathophysiological conditions such as intrauterine adhesion or thin endometrium [3, 49].

Biomaterial-based delivery of stem cells has been reported to be effective in promoting endometrium regeneration [50–53]. To our best knowledge, most previous stem cell researches in endometrium regeneration applied exogenous MSCs. However, the use of exogeneous MSCs faces multiple concerns including immunogenicity, oncogenicity and ethical issues [17]. Therefore, we sought to find an autologous resource of MSCs. ADMSCs are a kind of MSCs derived from adipose tissue and possess typical MSC properties. The stable resource and easy accessibility mean that ADMSCs could be conveniently acquired when needed, thus exempted from rigorous long-time preservation requirements. The autologous origin makes ADMSCs without immunogenic or ethical concerns. Moreover, single-cell analysis identified ADMSCs as a more controllable and advantageous option for stem cell therapy [54]. Collagen is among the most widely used biomaterials and shows favorable biocompatibility and biodegradation [55, 56]. As the first comprehensive research which integrated ADMSCs with biomaterials for endometrium regeneration, we observed better endometrium regeneration outcomes in the CS/ADMSC group, including higher endometrium thickness, more endometrial glands, less collagen deposition, denser microvessels and better fertility restoration.

Enough endometrial thickness is a prerequisite for successful embryo implantation and development as thin endometrium (≤7 mm) in humans is associated with low pregnancy rates [57]. Estrogen supplement therapy and other methods have been used in clinical practice to regenerate endometrium but with very limited success [58]. In our study, the sectional endometrial thickness and number of ki67^+^ proliferating cells of the CS/ADMSC group were higher than the other two groups. These results demonstrate that the CS/ADMSC can promote tissue growth after injury and thus retain tissue size. The predominance of ki67-positive cells in the CS/ADMSC group indicates that there is active proliferation even after a relatively long period (28 days). Endometrial glands also play an indispensable role in successful implantation [59]. The secretion of endometrial glands is crucial for stromal cell decidualization and lack of endometrial glands causes peri-implantation loss in mice [60]. Ethanol injury greatly reduced the number of endometrium glands while the gland number increased in the uterine horns with CS/ADMSC transplantation.

Endometrial fibrosis is one of the commonest pathological conditions causing uterine factor infertility and usually results in the formation of fibrotic adhesions [2, 61]. The major histological features of intrauterine adhesion include substitution of non-vascularized fibrotic tissue for endometrial stroma and loss of endometrial glands [61]. In the NR group, most endometrial area was replaced by collagen fibers (stained blue by Masson trichrome staining), mimicking the pathological features of intrauterine adhesion. Single CS implantation aggravated fibrosis, which might be due to the host foreign body reaction against solid implantations [35], and this phenomenon inspired us to search for and test more tissue-compatible materials and methods in future study [62, 63]. On the other hand, the CS/ADMSCs significantly ameliorated endometrial fibrosis, indicating that the ADMSCs exerted anti-fibrotic effects. TGFβ1 is the hub of fibrosis formation in pathological conditions such as intrauterine adhesion. TGFβ1 activates transformation of fibroblast to myofibroblasts, and it acts through smad2/3 phosphorylation to promote the expression of the ECM components such as Collagens I, III and V [44]. We performed *in vitro* experiments to investigate the mechanisms of the CS/ADMSCs anti-fibrotic effects. HESCs manifested fibrotic phenotype with increased expression of ECM components and myofibroblast transformation upon TGFβ1 stimulation (Fig. 5). The phosphorylation level of smad3 was upregulated by TGFβ1 but was brought down by CS/ADMSCs. This result indicated that the anti-fibrotic effect of CS/ADMSC was partially attributed to the suppression of smad3 phosphorylation.

Blood vessel networks are responsible for nutrient and oxygen supply, as well as removing metabolic wastes. Therefore, blood vessels play an essential role in tissue hemostasis. Sufficient angiogenesis after tissue injury is crucial for reconstruction of tissue organizations. The lack of blood supply would not only cause tissue hypoxia but also lead to fibrosis. Deposition of excessive ECM in extracellular space make the tissue stiffen and the tissue blood vessels would be obstructed in return [64]. Therefore, promoting angiogenesis is a major target in tissue repair [65]. Our results showed that the endometrium of the CS/ADMSC group exhibited the highest vessel density, while the endometrium in the other two groups were mainly fibrotic tissue with few vessels. Tube formation assay is widely used to evaluate the angiogenic effects *in vitro*. In the presence of CS/ADMSC, HUVECs exhibited high tube formation capacity. These results demonstrate the CS/ADMSC play an important role in promoting angiogenesis during endometrial repair.

Macrophages are the principal immune cells in the tissue inflammation microenvironment. After tissue injury, the coordination of inflammation and regenerative processes is essential for functional recovery [37, 39]. The phenotype switch of macrophages is a vital step in this coordination. Though the nomenclature of macrophage phenotypes can be complicated, a simplified M1/M2 dichotomy of macrophage phenotypes is widely accepted [40]. The M1 macrophages, also referred as classically activated macrophages are pro-inflammatory, expressing CD86 and secreting multiple pro-inflammatory cytokines, while the alternatively activated M2 macrophages are anti-inflammatory, expressing CD163 and secreting pro-regenerative cytokines. Here we observed that macrophages were recruited into the endometrium. Without treatments or with only collagen scaffold, the macrophages exhibited mainly pro-inflammatory M1 phenotype and ignited prolonged inflammation up to 7 days after tissue damage (Fig. 4). Although early inflammation after tissue damage is considered necessary for wound healing, excessive and prolonged inflammation could lead to disastrous consequences such as insufficient tissue proliferation, poor angiogenesis and abnormal fibrosis [37]. Our results showed that the CS/ADMSC implantation inhibited excessive M1 macrophage polarization and enhanced M2 polarization *in situ*, thereby suppressing local inflammatory microenvironment to favor endometrial regeneration. Flow cytometry also demonstrated CS/ADMSC suppressed M1 polarization (Fig. 4E and F). Cytokines secreted by M1 or M2 macrophages are also crucial mediators of local inflammation. IL1, IL6 and TNFα were pro-inflammatory cytokines mostly secreted by M1 macrophages and are reported to influence tissue regeneration [66, 67]. When stimulated by LPS, CS/ADMSC preconditioned macrophages expressed lower levels of all these three pro-inflammatory cytokines. We also observed discrepant levels of IL10 expression in the preconditioned and non-preconditioned macrophages after LPS stimulation, but the difference was relatively slight. Another M2-related cytokine TGFβ did not manifest disparity in LPS-stimulated macrophages, but arginase 1 was remarkably higher in the preconditioned macrophages even after LPS stimulation. These results indicated that CS/ADMSCs had robust anti-inflammatory effect by halting M1 polarization and preventing secretion of pro-inflammatory cytokines.

From an overall perspective, tissue regeneration involves multiple processes. Rapid closure of wound surface provides an early protection to the injured tissue, and the following robust proliferation is the cornerstone for maintaining original tissue size [68]. On top of that, guiding tissue cells to the right differentiation fates cannot be neglected [69]. For example, uncontrolled myofibroblast transformation would result in tissue fibrosis. Moreover, fibrosis, inflammation and poor angiogenesis are interrelated. Our study showed that the CS/ADMSC manifested multifaceted regenerative effects that were evidenced by *in vitro* and *in vivo* experiments and these results provided significant pre-clinical proof for our future clinical study using CS/ADMSC for endometrium repair.

## Conclusion

As the first study applying ADMSCs in endometrium regeneration, we constructed an ADMSCs composited collagen scaffold (CS/ADMSC) and proved its regenerative and immunomodulatory effects by *in vitro* and *in vivo* experiments; intrauterine implantation of CS/ADMSCs preserved fertility in a rat model. A transcriptome analysis revealed that the CS/ADMSC enriched biological processes involved in ECM components and organization, cell proliferation and wound healing in endometrial cells. Autologous ADMSCs hold great translational potential for regenerative medicine. Our study provided good evidence for the use of autologous ADMSC in subsequent clinical studies.

## Supplementary Material

rbad080_Supplementary_Data

## Data Availability

Data would be made available on request.
