# Supplementary material for: A construct of adipose-derived mesenchymal stem cells—laden collagen scaffold for fertility restoration by inhibiting fibrosis in a rat model of endometrial injury
Source: Regen Biomater. 2023 Sep 7;10:rbad080. doi: 10.1093/rb/rbad080 (PMC10551231; doi:10.1093/rb/rbad080)
Supplement: rbad080_Supplementary_Data [file rbad080_supplementary_data.docx]

**Supplement Information**

**A construct of adipose-derived mesenchymal stem cells–laden collagen scaffold for fertility restoration by inhibiting fibrosis in a rat model of endometrial injury**

**Authors:**

Yangyang Dai^#a,b,c^, Liaobing Xin^#a,b^, Sentao Hu^#a,c^ , Shiqian Xu^a,b^,Dong Huang^a,b^, Xiaoying Jin^a,b^, Jianmin Chen^a,b^, Rachel Wah Shan Chan^d,e^, Ernest Hung Yu Ng^d,e^, William Shu Biu Yeung^d,e^, Lie Ma*^a,c^, Songying Zhang*^a,b^

**Affiliations:**

^a^Assisted Reproduction Unit, Department of Obstetrics and Gynecology, Sir Run Run Shaw Hospital, Zhejiang University School of Medicine, 310016 Hangzhou, China

^b^Key Laboratory of Reproductive Dysfunction Management of Zhejiang Province, Hangzhou, China

^c^MOE Key Laboratory of Macromolecular Synthesis and Functionalization, Department of Polymer Science and Engineering, Zhejiang University, Hangzhou 310027, China

^d^Department of Obstetrics and Gynaecology, School of Clinical Medicine, LKS Faculty of Medicine, The University of Hong Kong, Hong Kong SAR, 999077, China

^e^Shenzhen Key Laboratory of Fertility Regulation, The University of Hong Kong Shenzhen Hospital, Shenzhen, 518000, China

*Corresponding author: [zhangsongying@zju.edu.cn](mailto:zhangsongying@zju.edu.cn); [liema@zju.edu.cn](mailto:liema@zju.edu.cn)

^#^These authors contributed equally to this research.

**FigureS1.** Cytocompatibility of the collagen scaffold：CCK8 absorbance reveal that CS have good cytocompatibility.

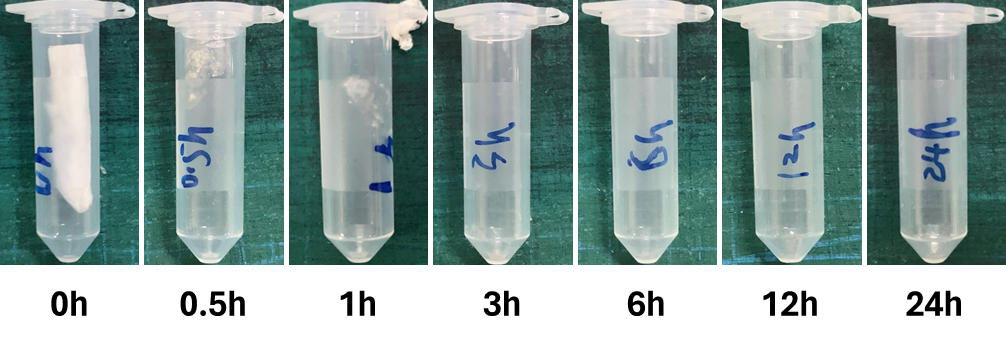


**FigureS2**: In vitro biodegradation of the collagen scaffold at a collagenase concentration of 0.1U/mL. The CS were completely degraded within 12 hours. In vivo degradation method: Dry CS was cut into small pieces and weighed at the precision of 0.01mg (w_cs0_); dry clean centrifugation tubes were also weighed (w_tube_). CS pieces were put in the tubes and collagenase in PBS (0.1U/mL) was added to the tubes, then the tubes were placed in a 37℃ shaker for different periods as labeled (triplet at each time point). Digestion was terminated by centrifugating the tubes (13000g 5min) and discarding the supernatant. The CS residuals in the tubes were washed 3 times with ddH_2_O in the tube and then dried at 80℃ for 12 hours. The total weight of the dry tube and CS residual was weighed (w_tube+residual_). Degradation percentage was calculated by the following formula:

$degradation percentage=\left( w_{cs0}-(w_{tube+residual}-w_{tube}) \right)/{w_{cs0}\times100\%}$.


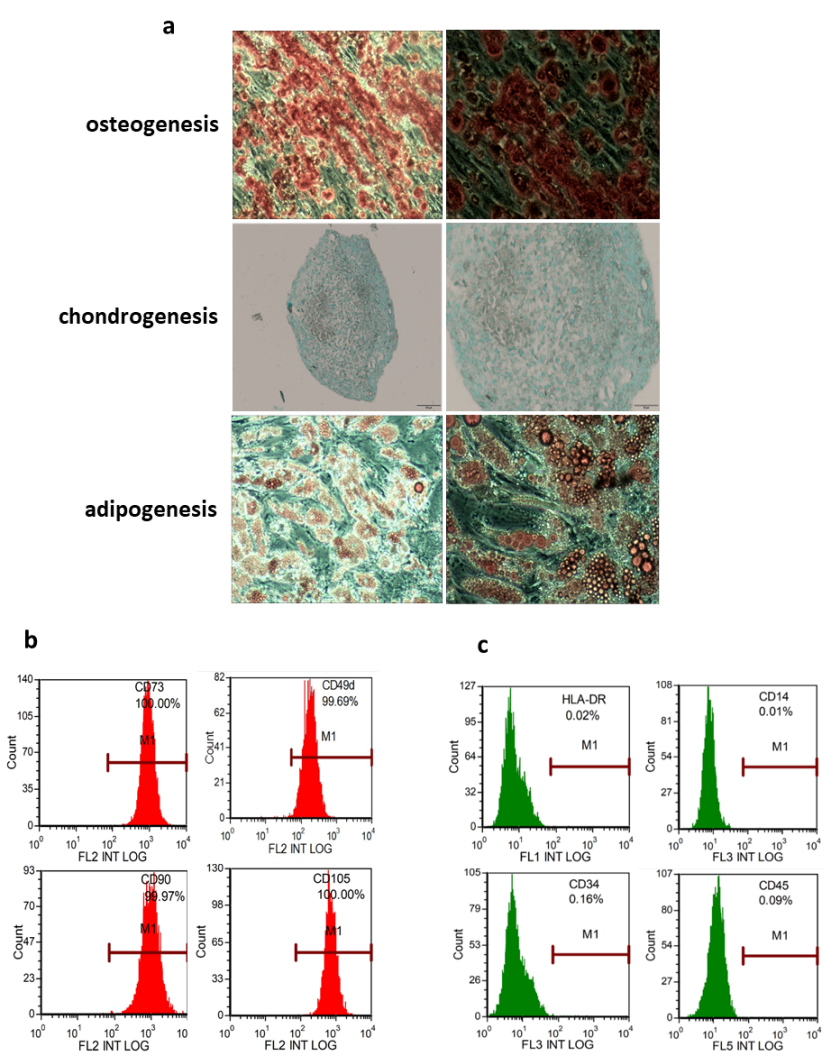


**FigureS3**. Characterization of ADMSCs: a) ADMSCs possess multilineage differentiation potentials; b) and c) ADMSCs express signature surface markers


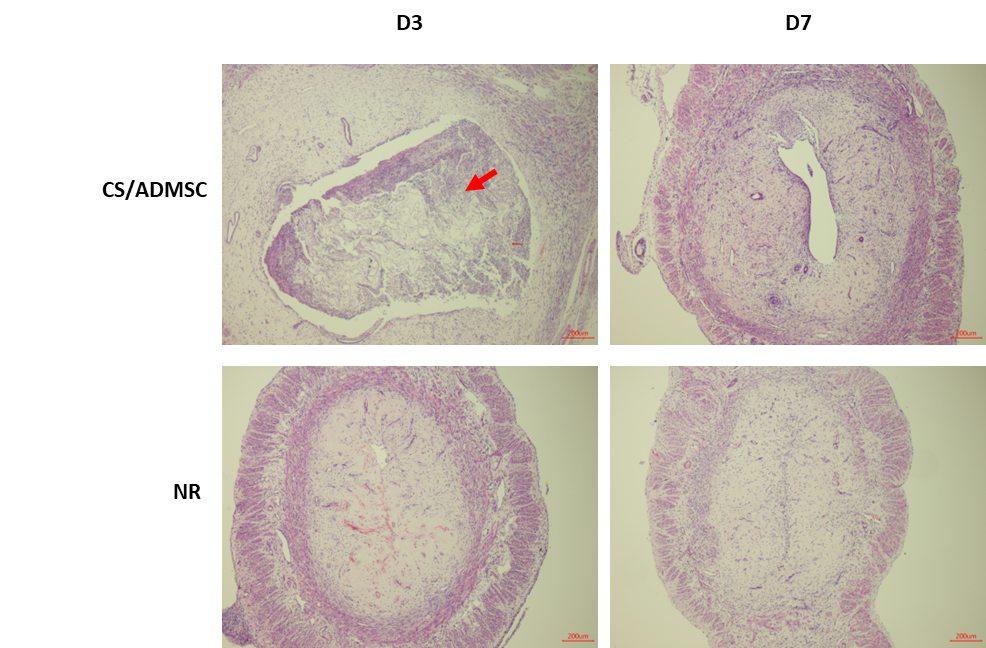


**FigureS4**. in vivo observation of CS/ADMSCs. CS/ADMSC would not be visually distinguished within 1 week in rat uterus, red arrow indicates the undegenerated CS/ADMSC on Day 3.


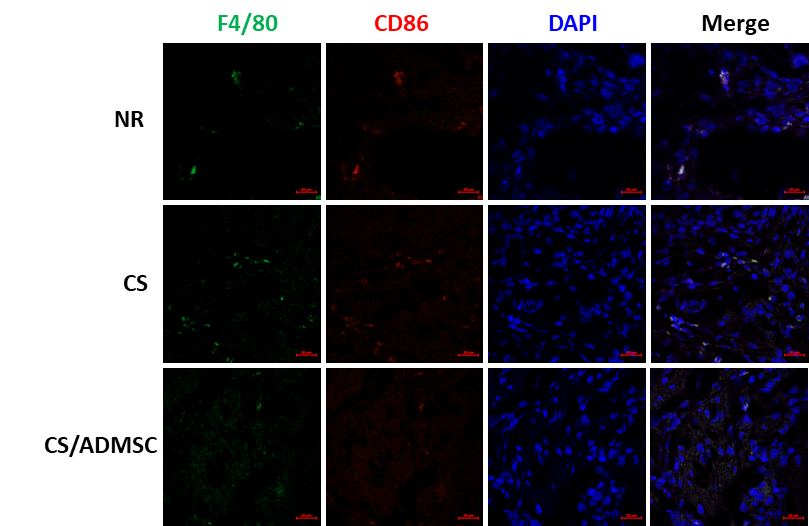


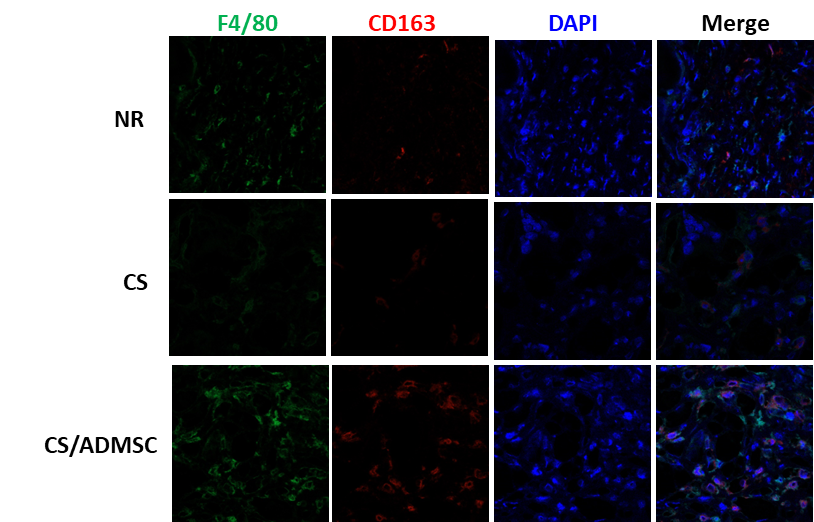


**FigureS5**. immunofluorescence of M1 and M2 macrophages on Day 3.

**Table S1**. Real time qPCR primers (H-and M- refer to human and mouse separately)

| Target name | Forward primer | Reverse primer |
| --- | --- | --- |
| H-COLA1 | CAAGAGTGGTGATCGTGGTGA | GGGAGACCGTTGAGTCCATC |
| H-αSMA | CTATGAGGGCTATGCCTTGCC | GCTCAGCAGTAGTAACGAAGGA |
| H-Fibronectin | AGGAAGCCGAGGTTTTAACTG | AGGACGCTCATAAGTGTCACC |
| H-Smad3 | TGGACGCAGGTTCTCCAAAC | CCGGCTCGCAGTAGGTAAC |
| H-SOX2 | CTCGTGCAGTTCTACTCGTCG | AGCTCTCGGTCAGGTCCTTT |
| H-Nanog | AAGGTCCCGGTCAAGAAACAG | CTTCTGCGTCACACCATTGC |
| H-OCT4 | CTTCGCAAGCCCTCATTTC | GAGAAGGGAAATCCGAAG |
| H-GAPDH | CGGAGTCAACGGATTTGGTCGTAT | AGCCTTCTCCATGGTGGTGAAGAC |
| M-IL-1 | GAAATGCCACCTTTTGACAGTG | TGGATGCTCTCATCAGGACAG |
| M-IL-6 | ACAAAGCCAGAGTCCTCAGAGAG | TTGGATGGTCTTGGTCCTTAGCCA |
| M-TNFα | CCCTCACACTCAGATCATCTTCT | GCTACGACGTGGGCTACAG |
| M-IL-10 | GCTCTTACTGACTGGCATGAG | CGCAGCTCTAGGAGCATGTG |
| M-TGFβ | GTGCGGCAGCTGTACATTGACTTT | TGTGTTGGTTGTAGAGGGCAAGGA |
| M-Arginae1 | GCTCAGGTGAATCGGCCTTTT | TGGCTTGCGAGACGTAGAC |
| M-GAPDH | AGGTCGGTGTGAACGGATTTG | GGGGTCGTTGATGGCAACA |
